# Egg Quality, Yolk Fatty Acid Profiles from Laying Hens Housed in Conventional Cage and Cage-Free Production Systems in the Andean Tropics

**DOI:** 10.3390/ani14010168

**Published:** 2024-01-04

**Authors:** Roy Rodríguez-Hernández, Iang Schroniltgen Rondón-Barragán, Edgar O. Oviedo-Rondón

**Affiliations:** 1 Poultry Research Group, Faculty of Veterinary Medicine, University of Tolima, Altos the Santa Helena, A.A 546, Ibague 730006299, Colombia; royrodriguezh@ut.edu.co (R.R.-H.); isrondon@ut.edu.co (I.S.R.-B.); 2 Immunobiology and Pathogenesis Research Group, Faculty of Veterinary Medicine, University of Tolima, Altos the Santa Helena, A.A 546, Ibague 730006299, Colombia; 3 Prestage Department of Poultry Science, North Carolina State University, 2711 Founders Drive, Scott Hall O-239, Raleigh, NC 27695, USA

**Keywords:** housing, welfare, quality, stress

## Abstract

**Simple Summary:**

The poultry industry is considered relevant to global food security. Eggs and chicken meat remain a crucial, essential source of human nutrition. Currently, consumers of animal-origin food products are concerned about animal welfare in intensive production systems, the harmful effects on animal health, and the quality and safety of products; the conventional cage (CC) housing system is one of the most common egg production systems in several countries. This study compared the egg quality parameters produced by laying hens in two egg housing systems, CC and Cage Free (CF), under commercial conditions up to the 82nd week (wk) in the Andean tropics. The results suggest that the conditions of the housing systems evaluated can affect internal and external egg quality parameters.

**Abstract:**

Egg consumers worldwide have increased their concerns about laying hens’ welfare and its impact on final egg product quality. This study compared the egg quality parameters under the conventional cage (CC) and cage-free (CF) egg production systems in the tropics. The study was conducted on a commercial farm in Colombia using Hy-Line Brown pullets, reared under the same conditions for the first 15 wks. At 16 wks, the hens were distributed into two housing systems, CC and CF, on the same farm. The hens were fed the same diet for each phase in both systems and feed intake varied slightly. Egg samples were collected every six wks, from 22 to 82 wks of age. A total of 3960 eggs were analyzed at 11 sampling times. Parameters such as albumen height, egg weight, yolk color, eggshell thickness, eggshell strength, and Haugh units were determined using a DET-6000 machine. At 22 and 82 wks, screening for *Salmonella* spp. status was conducted using environmental and egg samples. Additionally, at 34, 64, and 82 wks, yolk samples were obtained for fatty acid profiles and crude protein (CP) analysis. The data were analyzed in a completely randomized block design with repeated measures (11 times): mean separation by Student’s *t*-test yolk pigmentation, Haugh Units, and albumen height (*p* < 0.001) were higher in the CF compared with the CC between 38 and 69 wks of age, and eggs at 63 and 82 wks (*p* < 0.05) were heavier in the CF compared to the CC. Likewise, eggs from the CC had better eggshell strength from 57 to 82 wks. In the egg yolk fatty acid profile at the 34th wk, the pentadecanoic, palmitic, and heptadecanoic acids had higher concentrations in the CF systems than the CC. At the 64th wk, the egg yolk fatty acids—lauric, myristic, and heptadecanoic—had higher concentrations in the CF; likewise, at the 82nd wk, egg yolks from the CC had higher concentrations of lauric, heptadecanoic, and nervonic fatty acids than the CF. The eggs and environmental samples were negative for *Salmonella* spp. throughout the whole production phase. These results indicated that the production system might impact internal and external egg quality measures, potentially due to various stressors, including environmental factors or behavior restrictions.

## 1. Introduction

The table egg industry is already considered a relevant and sustainable food production system [1]. It continues to play an increasing role in global food security, given its ability to supply affordable, high nutritional value protein and essential micronutrients for the human population [2,3]. However, providing high-quality food for the increasing world population and improving the efficiency of protein production sustainably is a challenge for animal agriculture [3]. Therefore, in response to population growth and living standards, the poultry sector has increased its production systems to supply global food demand [4]. Notwithstanding, the conventional cage (CC) housing system is one of the most common, which allows enhanced egg production with high hen populations housed in small areas, and it represents 90% of global egg production [5,6,7].

Nevertheless, the CC system has been questioned due to the restriction of movement that limits natural behaviors and increases cloaca pecking, aggression, and plumage damage, which can generate changes in the transcription of genes related to productivity, immunity, and metabolism [8,9]. Currently, consumers of poultry products are increasingly aware of the egg production systems and have strong opinions on hen welfare [10]. Therefore, to guarantee the welfare of their hens, some producers are changing to a cage-free (CF) system with conditions perceived as being better for animal welfare, which now represents 10% of egg production globally and is rapidly expanding [7].

In poultry production, birds can be susceptible to diverse stressors, including adverse environments, transportation, and infection, among others [11]. The deleterious effect of stress on egg quality includes a decrease in egg weight, shell thickness, yolk weight, and low albumen weight, possibly due to the reduction in feed intake, changes in gene expression, and other neuroendocrine responses in birds [11,12,13,14]. Housing systems can impact all these factors, and considerable research has been developed in the past 30 years to evaluate its effects on egg quality. However, controversial results have been found among studies evaluating the effects of egg production systems on egg quality, performance, foot injuries, and feather scores between hens housed in CC, enriched cages, and non-restrictive production systems. These differences could be due to a lack of standardization in the methodologies [15,16,17,18]. Nevertheless, several factors can influence egg quality, such as the genetics and age of the laying hen, the season or prevalent environmental conditions, nutrition, and health conditions. Some authors suggest that all factors should be studied simultaneously to verify the effect of different housing systems under the same experimental conditions and understand how each will influence egg quality [15]. However, such a study remains a major methodological challenge.

However, the housing, hen and egg management, and environmental conditions in the tropical Andes differ from European, North American, and Brazilian production systems. For example, since the weather in the Andean Tropics is not as severe as in Europe or North America, no poultry company invests in total environmental control or complete wall and ceiling thermal insulation. Flock body weight gain is controlled more frequently, and grading selection by weight is more common in the Andean region than in other countries where labor is more expensive. Eggs are not washed or refrigerated in the Andean region as in other countries. Consequently, research under these conditions is needed to quantify the impact of these housing systems on egg quality.

This research compared the internal and external egg quality, including fatty acids and protein in yolk, from hens housed in two egg production systems, CC and CF housing systems, until the 82nd wk, and the possible effect of the egg production system on the presence of *Salmonella* spp.

## 2. Materials and Methods

### 2.1. Study Population and Sample Collection

The study was conducted on a commercial farm located in Ibague city, department of Tolima, in the central area of Colombia, between 02°52′59″ and 05°19′59″ north latitude, and 74°24′18″ and 76°06′23″ west longitude, at 1250 m above sea level, with an average temperature of 25°C. The Tolima district is located between the central and eastern mountains of the Colombian Andes.

Under commercial conditions, 60,000 one-day-old Hy-Line Brown pullets were placed in cages (Modular Manure Belt Brood Grow) with an area of 76.22 × 66.05 m and a density of 16 pullets/cage (315 cm^2^/bird). The pullets were reared with the same sanitary conditions, management, and feed program up to 15 wks of age. Later, the same pullets were transferred into two different housing systems, CC and CF, on the same farm, up to 82 wks of age. A total of 45,000 hens were housed in a CC system in a pyramidal multi-deck battery of vertical cages in Californian-type facilities (40 × 40 × 40 cm). Each battery had four floors, nipple drinkers, and the house had a cooling panel ventilation system. In this house, we placed four hens per cage (450 cm^2^/hen).

For this study, 720 hens were evaluated in 15 replicates of 12 cages each (48 birds/replica) for a total of 180 cages assessed in the CC system. The CF system evaluated was an aviary type; it had a deep litter floor using rice husks in conventional houses, open sheds (mesh-sided), and natural ventilation (wind only). Each house had communal nest boxes, which are wooden structures with two levels, each level with 10 nests, measuring 40 cm × 40 cm × 40 cm (5 hens/nest), with a 4 m perch per level. The 14,850 hens were distributed in two poultry houses with a density of 1111 cm^2^/bird. These poultry houses were divided into 15 rooms and used as replicas for the CF system, each replica with 990 hens/room. Water and feed were offered, and a lighting program of 14L:10D was used.

The environmental temperature and humidity were recorded hourly using a Hobo data logger (Onset Corp., Cape Cod, MA, USA) for 26 wks (24 h/7 days), obtaining 10,145 data points. Temperatures varied slightly, with a CF mean and SD of 24.45 ± 2.80 °C and CC mean of 24.7± 2.81 °C. The humidity varied by 7% between production systems throughout the study. The diets were formulated based on the recommendations of the Hy-Line Brown Layer Management Guidelines (Table 1), and the hens were fed the same diet in both systems. However, the feed intake varied slightly between the treatments, and 720 hens (48 birds/replicate) were weighed per production system each wk and showed statistical differences (Table 2). Health and nutritional management were carried out according to the poultry company policies.

### 2.2. Egg Quality Measurement

Egg quality samples were collected approximately every six wks, from the 22nd to 82nd wks of age. In the CC system, one egg per cage, twelve per replicate, from fifteen replicates for a total of 180 eggs were collected in each wk sampled. Twelve eggs per room from fifteen replicates were obtained in the CF system for a total of 180 eggs evaluated in the CF system. Therefore, 360 eggs were obtained to assess egg quality in both production systems every six wks. The egg quality assessment was performed using a DET 6000 digital egg tester (Nabel Co. Ltd., Kyoto, Japan). The eggs were weighed (g) and cracked. Then, albumen height (mm), yolk color (Yolk Fan™, color score: 1–16), eggshell thickness (mm), and eggshell strength (kgf) were determined, and Haugh units were calculated utilizing HU formula HU = 100 × log (h − 1.7w^0.37^ + 7.6). HU: Haugh unit; h: albumen height (millimeters); w: egg weight (grams) [19].

### 2.3. Yolk Fatty Acid Analysis

Eggs collected from hens at 34, 64, and 82 wks of age were taken for fatty acid analysis. Two pools of five egg yolks from each wk (100 g) were taken for FA analyses.

The fatty acid analysis was conducted by obtaining and quantifying the methyl ester using gas chromatography with flame-ionization detection ((GC-FID) AT 6890N gas chromatograph (Agilent Technologies, Palo Alto, CA, USA)) following the methods according to [20,21]. The reference standard was AccuStandard, Inc.’s 37 Component FAME Mix (125 Market Street, New Haven, CT 06513, Cat. No. FAMQ-005). DB-23 (J & W Scientific, Folsom, CA, USA) [50%-cyanopropyl-poly (methylsiloxane), 60 m × 0.25 mm × 0.25 µm] was the column utilized in the analysis. The injection was performed in split mode (50:1) (Viny = 2 μL).

### 2.4. Yolk Protein Analysis

Egg yolk pools (five yolks) at 34, 64, and 82 wks were analyzed for total nitrogen content using the Kjeldahl method, and the conversion factor from nitrogen to protein was 6.25. Ash content was determined by holding samples at 550 °C for 8 h, and pH was measured by a glass electrode using a 1:2 (egg:water) ratio.

### 2.5. Salmonella Flock Status

Considering the zoonotic importance of the *Salmonella* status of a layer flock and the impact on egg quality, only this microbiological parameter was evaluated. At 22 and 82 wks, the presence of *Salmonella* spp. in the flocks and eggs was determined using CC and CF systems with environmental samples (cage, egg belt, boot-swabs) in the CC and egg samples (nest surface, perch, boot-swabs, wire mesh) in the CF. For environmental samples, two boot and two surface swabs from each production system were collected in sterile bags (Nasco^®^, Fort Atkinson, WI, USA).

Additionally, three eggs were pooled for every sampling time for each of the 15 replicates (15 pools from 45 eggs) per production system. Their egg content and eggshells were analyzed independently using bacteriological procedures. Samples obtained were processed in the Veterinary Diagnostic Laboratory of the University of Tolima for microbiological culture to isolate *Salmonella* spp. by specific standard procedures. Briefly, the environmental swabs were mixed with 25 mL of buffered peptone water (BPW), and the shells of three eggs of each of 15 replicates (pools) per production system were mixed with 250 mL of BPW in a Stomacher bag; the eggs’ contents were homogenized for 1 min and mixed with BPW at a 1:1 ratio. Environmental, eggshell, and egg content samples were incubated at 37 °C ± 1 °C for 24 h. Then, in the pre-enrichment step, 0.1 mL of medium was inoculated into 10 mL of Rappaport Vassiliadis broth (Oxoid, Basingstoke, Hampshire, UK) and incubated at 41.5 °C ± 1.0 °C for 24 h. Then, 0.1 mL of a second aliquot of the pre-enriched medium was inoculated into 10 mL of tetrathionate broth (Oxoid, Basingstoke, Hampshire, UK) and incubated for 24 h at 37 °C ± 1 °C. Later, an aliquot of the tetrathionate and Rappaport Vassiliadis cultures was inoculated into xylose lysine deoxycholate and MacConkey and incubated at 37 °C ± 1.0 °C for 24–48 h [22,23].

### 2.6. Statistical Analysis

The data were analyzed for descriptive statistics in a completely randomized block design with repeated measures and mean separation by Student’s *t*-test using GraphPad Prism v10 for MacOS software (LaJolla, CA, USA). The experimental units were 15 replicates per production system. In the CC, there were 12 cages of 4 hens each for each of the 15 replicates. At the same time, the CF had 15 rooms located in two houses, each with 990 hens and similar housing density. Subsamples of these replicates were averaged before statistical analysis. In all cases, *p* < 0.05 was considered statistically significant.

## 3. Results

### 3.1. Egg Quality

#### 3.1.1. Egg Weight

**Figure 1:**
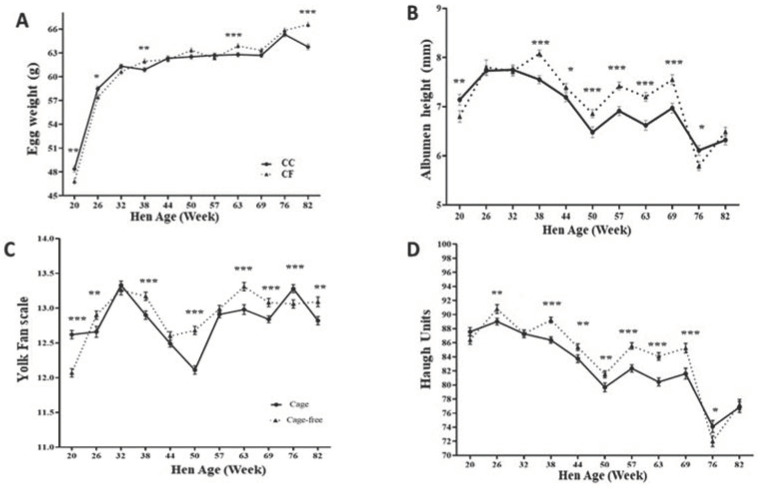
Comparison of egg weight (**A**), albumen height (**B**), yolk pigmentation (**C**), and Haugh units (**D**) from conventional cage (CC) and cage-free (CF) housing systems collected from 20 to 82 wks of age. The data are presented as the means ± SEM (*n* = 180/system/week). * *p* < 0.05; ** *p* < 0.01; *** *p* < 0.001 (Student’s *t*-test).

A total of 3960 eggs were analyzed at 20, 26, 32, 38, 44, 50, 57, 63, 69, 76, and 82 wks of age (11 sampling times). For the eggshell quality measurements, differences in egg weight were observed at the 20th (*p* < 0.01) and 26th wks (*p* < 0.05). The eggs from hens housed in the CC were heavier than in the CF system. At the 38th, 63rd, and 83rd wks, the eggs from hens in the CF system were heavier than eggs from the CC (*p* < 0.01), respectively (Figure 1A).

#### 3.1.2. Internal Quality

In the same way, at 20 wks of age, albumen height was higher in eggs produced from hens housed in the CC system (*p* < 0.01). But, from wk 38 up to the 69th wk, the albumen height was higher in the eggs produced in the CF than the CC (*p* < 0.05 and *p* < 0.001) (Figure 1B). Similar results were obtained in yolk pigmentation. Eggs produced in the CC showed a higher pigmentation only at the 20th and 76th wks in the CC (*p* < 0.001). Nevertheless, eggs from hens housed in the CF system exhibited a higher yolk pigmentation than the CC (*p* < 0.01) at 26, 38, 50, 63, 69, and 82 wks.

The Haugh unit index in eggs from the CF system was higher in most wks sampled compared with the CC. At 26, and from 38 up to 69 wks of age (*p* < 0.001), the Haugh units significantly decreased with the increasing age of laying hens. Likewise, eggs from the CC had better eggshell strength at 57 to 82 wks.

#### 3.1.3. External Quality

**Figure 2:**
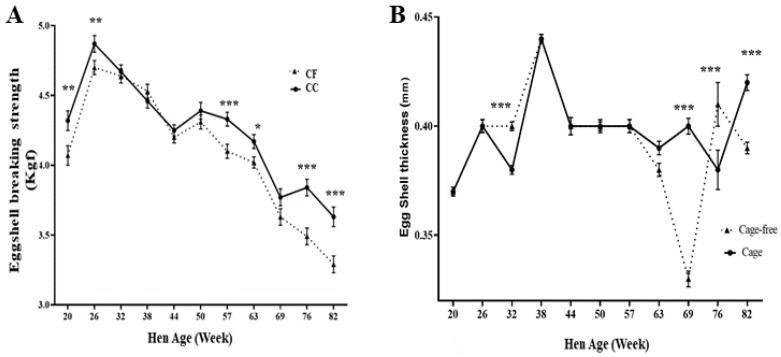
Comparison of eggshell resistance (**A**) and eggshell thickness (**B**) from conventional cage (CC) and cage-free (CF) housing systems of experimental 20–82nd-week data are presented as the means ± SEM (*n* = 180/system/week). * *p* < 0.05; ** *p* < 0.01; *** *p* < 0.001 (Student’s *t*-test).

The evaluation of eggshell-breaking strength indicated that eggs were more resistant to rupture in the CC than in the CF (*p* < 0.05). Still, similar results were observed at wks 32, 38, 44, and 50. The eggshell thickness showed differences (*p* < 0.001) at 32, 69, 76, and 82 wks (Figure 2), but they were variable between the two production systems. The significant drop in eggshell thickness at week 69 could be due to a heat wave that caused more heat stress in this housing system. The CF system was not a controlled environment, and in weeks 68 and 69 these hens suffered heat stress, evidenced by a higher temperature and humidity index in the CF system than in the CC. Heat stress increases panting, causing alterations in the acid–base balance and consequently reducing eggshell mineralization. This effect was not observed seven weeks later in the subsequent sampling at 76 weeks of age, and the eggshell mineralization was better.

### 3.2. Yolk Fatty Acid Analysis

**Figure 3:**
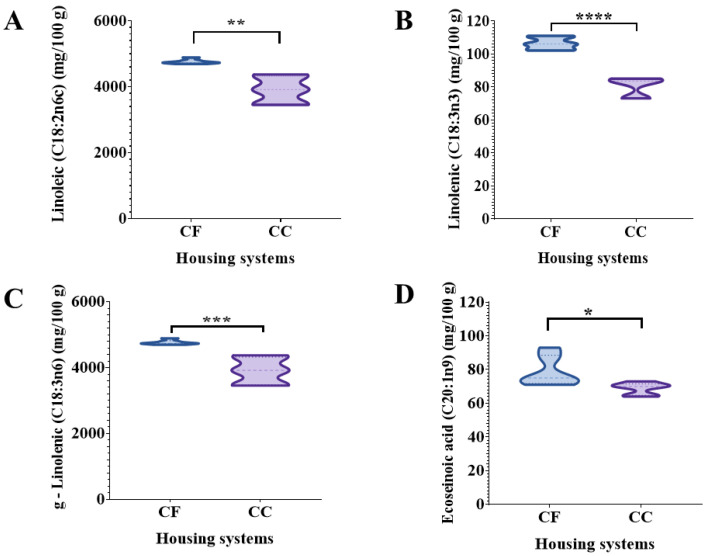
Comparison of polyunsaturated fatty acid concentrations (mg/100 g) in egg yolks at the 34th week from hens housed under the conventional cage (CC) and cage-free (CF) systems. Data are presented as the means ± SEM (*n* = 5 pools/system/week). (**A**) linoleic acid; (**B**) linolenic acid; (**C**) g-linolenic acid; (**D**) eicosenoic acid. * *p* < 0.05; ** *p* < 0.01; *** *p* < 0.001; **** *p* < 0.0001 (Student’s *t*-test).

**Figure 4:**
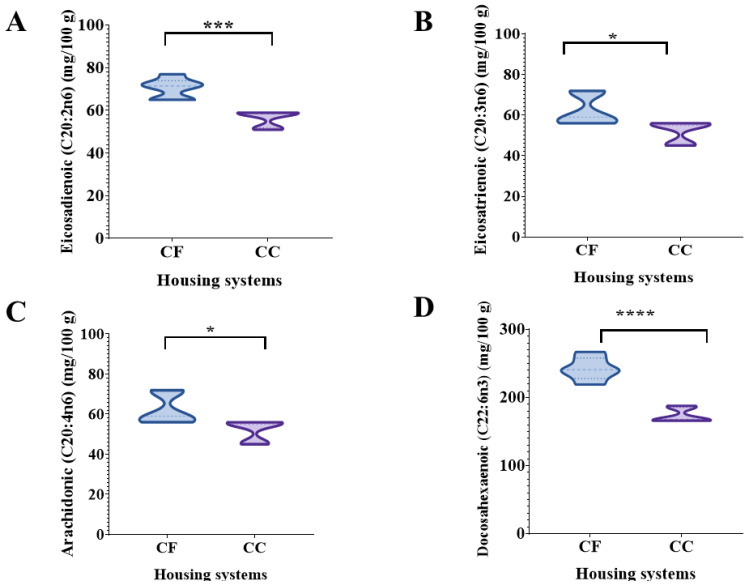
Comparison of polyunsaturated fatty acid concentrations (mg/100 g) in egg yolks at wk 34 from hens housed under the CC and CF systems. Data are presented as the means ± SEM (*n* = 5 pools/system/week). (**A**) eicosadienoic acid; (**B**) eicosatrienoic acid; (**C**) arachidonic acid; (**D**) docosahexaenoic acid. * *p* < 0.05; *** *p* < 0.001; **** *p* < 0.0001 (Student’s *t*-test).

In the 34th wk, the concentration of saturated fatty acids in yolk samples differed (*p* < 0.001) between the two systems. Pentadecanoic, palmitic, and heptadecanoic acids had a higher concentration in egg yolks from the CF systems than the CC. However, stearic acid had a higher concentration in the CC systems than the CF (Table 3). The concentrations of polyunsaturated fatty acids (PUFAs), linoleic, linolenic, g-linolenic, eicosenoic, eicoisadienoic, eicosatrienoic, arachidonic, and docosahexaenoic acids, were higher in the egg yolks in the CF system than the CC (Figure 3 and Figure 4). At the 64th wk, the lauric, myristic, and heptadecanoic acids had higher concentrations in egg yolks in the CF systems. Still, stearic and palmitic acids were higher in the eggs from the CC. Palmitoleic and oleic acids, which are monounsaturated fatty acids, at the 64th wk showed a higher concentration in the CC. Still, the eicosenoic acid concentration was higher in the eggs from the CF system than in the CC, likewise, the linoleic and eicosatrienoic PUFAs. At the 82nd wk, egg yolks from hens housed in the CC had higher concentrations of lauric, heptadecanoic, and nervonic fatty acids than the CF (Table 3).

### 3.3. Yolk Protein Analysis

Egg yolk pools obtained at 34 and 64 wks from hens housed in CF and CC systems did not show statistical differences between production systems. However, at the 82nd wk, the CP was higher in egg yolks produced in the CF system (Table 4).

### 3.4. Salmonella Status

After the onset of lay at the 22nd wk and at the flock depopulation at the 82nd wk, no *Salmonella* spp. were isolated from environmental and egg samples.

## 4. Discussion

The CC is still the most common housing system for laying hens worldwide [15,24], but it has been criticized for impeding some hens’ natural behaviors. This situation has promoted the transition to CF systems, which are currently considered to offer better animal welfare conditions [25,26]. Currently, a high percentage of egg consumers in many countries care about hens and tend to purchase more eggs from hens not kept in cages [27]. However, the growing demand for CF eggs in many regions of the world causes challenges to producers transitioning from CC to CF systems, due to higher costs and biosecurity issues [28]. Nevertheless, housing seems to impact egg production and quality traits [25].

The quality of eggs could be affected by multiple internal and external factors such as genes, nutritional aspects, age of hens, management, and housing systems [24,25,29]. In this study, eggs from hens housed in a CC collected at the 20th to 26th wks were heavier than those in the CF system. However, eggs from hens in the CF system collected at 38, 63, and 83 wks of age were heavier than the CC. Similar results were reported by Racevičiūtė-Stupelienė et al. (2023), who observed heavier eggs from hens housed in a barn at 28, 32, and 36 wks of age compared to eggs from the enriched cage system [29]. The results of the current experiment can be explained by the initial adaptation that pullets moved to the CF system had to make. In the production system described in this study, the pullets were raised in cages using nipple drinkers and were then moved to CF (barn floor) conditions using bell drinkers, perches, and the rice husk floor substrate. These changes modified the feed and water consumption at 16 and 17 wks, decreasing the bird’s body weight gain, which potentially affected the internal and external egg parameters at the start of the lay. In contrast, the pullets moved from rearing cages to the CC system were moved to an environment with similar space, feeders, and drinkers.

Therefore, in the present study, at the 20th wk, when the hens started laying eggs, the albumen height, yolk pigmentation, and Haugh units were better in the eggs produced from the hens housed in the CC system, but from wk 26 and in almost all the wks sampled, these parameters were better for the CF eggs than the CC eggs and decreased as the hens aged. Roberts and Chousalkar (2014) showed that the egg internal quality parameters—albumen height, Haugh unit, and yolk color—were better in the CC production system than in Free Range, and quality decreased with flock age, except for the yolk color [30]. On the other hand, it has been reported that small groups of hens present a high feed intake and less movement due to cage size. These factors can cause hens to produce heavier eggs initially [31]. Samiullah et al. (2017) found that internal egg quality was significantly affected by the housing system, had the highest values of albumen height and the Haugh units in the free-range (FR) system. In eggs produced in cages and barn production systems, the yolk color was darker than in the FR system [16].

In contrast, Şekeroğlu et al. (2014) did not find an effect of housing on the egg quality in the ATAK–S Brown Hybrid laying hen line [32]. Kucukkoyuncu et al. (2017) mentioned that the hen age affects egg weight more than the housing system [33]. However, the shell and yolk weights were significantly higher in FR eggs than in CC eggs. Likewise, Sokołowicz et al. (2018) reported that the housing system did not affect albumen height and Haugh units, but the yolk pigmentation was lower in hens housed on a floor litter system [34]. Nevertheless, Racevičiūtė-Stupelienė et al. (2023) did not find statistical differences between housing systems when comparing eggshell thickness identified throughout all trial periods [28]. This differed from the present study, where statistical differences were found in four of the sampled wks. The differences could probably be due to environmental conditions and different genetic lines.

In the nutritional egg quality, the fatty acid profile variations in yolk may be linked to differences in genetic line, feed composition, microbiota, and other factors affecting the yolk composition [35,36]. The physical parameters and fatty acid profiles of eggs have shown high variability among different production systems [36]. In previous studies, PUFAs n-6 (linoleic acid, arachidonic acid) were present in lower concentrations in the yolk of eggs produced in FR and organic systems than in CC systems [34,37]. Racevičiūtė-Stupelienė et al. (2023) found the egg yolks from enriched cage housing systems were 0.04, 0.25, and 0.13% higher in heptadecanoic (C17:0), arachidonic (C20:4 n-6), and eicosapentaenoic (C20:5 n-3) acids than barn-laid eggs [28]. However, in our study, the linoleic acid (C18:2n6c) in the egg yolk was higher by 18% and 11% in eggs from the CF system than in eggs from the CC at 34 and 64 wks. Likewise, the g-linolenic (C18:3n6) and linolenic (C18:3n) acids in the CF egg yolks were greater by 25% in the 34th week than in the CC eggs.

Linoleic acid is an essential PUFA that functions as a structural component to maintain a membrane fluidity of the transdermal water barrier of the epidermis, a source of energy and cell signaling [38]. Likewise, results reported by English (2021) for Black and Grey Australorps and Rhode Island Reds in FR systems showed higher content of linoleic acid in eggs produced under CF systems; however, the diet included vegetable peels, scraps, lay mash, leftover bread, raw goat’s and cow’s milk, grass, insects, and worms [7].

The diet can change the egg yolk fatty acid profile. However, in this study, hens in both the CC and CF received the same diet and varied only slightly in feed consumption. In addition, in eggs at the 82nd wk, the CP was higher in yolks from the CF system. Research reports that have found differences in fatty acid composition and protein differ with their dietary intake. Additionally, those studies could be affected by the age, genetics of the hens, and modification of feed ingredients [39,40,41].

Consequently, it is expected that differences in egg fatty acid composition, egg protein, and quality parameters are also related to changes in microbiota. It is possible that hens in CF systems, continuously exposed to different materials in the deep litter, such as rice husk, excreta, and soil, may have different microbiota than hens housed in CC. The gut microbiota can also be modified by physical exercise in CF hens [42,43].

On the other hand, egg microbiological characteristics are important for quality and food safety. The main concern with eggs is *Salmonella* contamination. The environmental and egg samples were negative for *Salmonella spp,* from the onset of laying at week 22 of age until flock depopulation at 82 weeks of age. Similar results were reported by Roberts and Chousalkar in 2014 [30]. However, the results of multiple investigations evaluating the influence of production systems on the prevalence and persistence of *Salmonella* in laying hens could differ due to variability in the methodologies used to detect the prevalence and the populations being studied in different countries. Some studies suggest the laying hen strain should be considered when deciding on the housing environment to choose [44,45]. Nevertheless, housing system-specific characteristics include hygienic status, air quality, litter, and droppings, thus increasing the risk of contamination [46]. Under commercial conditions, high hygienic protocols and good biosecurity are followed and contribute to the absence of *Salmonella* contamination [30,44]. *Salmonella* contamination in production systems is linked more to biosafety failures than the production system itself.

Our findings challenge us to further investigate the possible influence of production systems on the expression of genes associated with metabolism, egg quality, and the effect of the intestinal microbiome on egg quality. Consequently, more research is necessary to determine if the hens housed in the CF system, having more contact with diverse substrates (soil, rice husk), could acquire differential microbiota that allow better availability and absorption of nutrients from the feed, generating better internal and external quality of the egg. Then, it is necessary to perform a metagenomic analysis to determine if there is a difference in microbial diversity in the hen gastrointestinal tract.

## 5. Conclusions

In the present study, we observed that the CF system showed superior egg quality parameters, such as albumen height, Haugh units, and yolk color, to CC systems but showed similar egg weight. The CC eggs had better eggshell breakage resistance than the CF eggs. Likewise, the CF eggs had a higher concentration of PUFAs at 34 and 64 wks. Differences in these results can be observed in the first wk of laying, when the pullets have to adapt to a new environment, feeder, and drinker type. Therefore, based on the results of the 11 samplings during the production process, we can conclude that the production system affects the egg quality. Furthermore, the production system did not affect the *Salmonella* status in this study, suggesting that the presence of this bacteria may be linked to biosecurity breaches in any system. Finally, we concluded that better hen welfare could generate better egg quality.

## Figures and Tables

**Table 1:** Composition of laying hens diets used to the commercial company, based Hy-Line Brown guide.

Diet Composition	Peaking	Layer 2	Layer 3	Layer 4	Layer 5
Crude protein (g/day)	17	16.7	16	15.5	15
Metabolizable energy (kcal/bird/day)	320	325	310	305	305
Lysine (mg/day)	830	810	790	770	750
Methionine (mg/day)	415	405	395	385	375
Methionine + cysteine (mg/day)	746	728	710	692	673
Threonine (mg/day)	574	560	546	532	518
Ca (g/day)	3.8	4.00	4.2	4.4	4.6
P (mg/day)	438	419	384	355	331
P (digestible) (mg/day)	396	376	347	321	300
Na (mg/day)	180	180	180	180	180
Cl (mg/day)	180	180	180	180	180

**Table 2:** Feed intake and body weight (mean ± SEM) from hens housed in CC and CF egg production systems at 11 sampling times.

	Feed Intake Mean (g/day)		*p*-Value	Body Weight (g)		*p*-Value
Age/Hens	CC	CF		CC	CF	
(Weeks)						
20	100.1 ± 1.7	98.7 ± 0.4	0.0701	1822 ± 5.84	1690 ± 4.6	<0.0001
26	113.5 ± 0.0	120.2 ± 0.1	<0.0001	1961 ± 5.64	1812 ± 4.3	<0.0001
32	114 ± 0.0	119.3 ± 0.1	<0.0001	1979 ± 6.13	1870 ± 4.3	<0.0001
38	111.6 ± 0.1	119.1 ± 0.3	<0.0001	1929 ± 6.0	1868 ± 4.7	<0.0001
44	112.1 ± 0.0	120.3 ± 0.2	<0.0001	1921 ± 5.7	1890 ± 4.4	<0.0001
50	112.7 ± 0.0	118.6 ± 0.0	<0.0001	1962 ± 6.6	1864 ± 5.0	<0.0001
57	113.4 ± 0.0	114.8 ± 0.9	>0.9999	1954 ± 7.2	1846 ± 5.3	<0.0001
64	114 ± 0.0	117.6 ± 1.1	0.0565	1973 ± 6.2	1925 ± 4.8	<0.0001
69	112.1 ± 0.0	118.9 ± 0.5	<0.0001	1976 ± 7.0	1918 ± 4.7	<0.0001
76	105.8 ± 0.0	113.5 ± 2.0	0.0182	2029 ± 7.2	1926 ± 5.8	<0.0001
82	111.1 ± 0.1	114.9 ± 0.6	0.0023	1996 ± 6.8	1931 ± 7.3	<0.0001

**Table 3:** Comparison of the saturated and polyunsaturated fatty acid concentrations (mg/100 g) in egg yolks at 34, 64, and 82 weeks from hens housed under the CC and CF. Data are presented as the means ± SEM (*n* = 5 pools/system/week).

Fatty Acid		Week	CF	CC	*p*-Value
Pentadecanoic acid	C15:0	34	18.83 ± 0.4	14.83 ± 0.4	<0.0001
Palmitic acid	C16:0	34	7326.5 ± 175.8	6829 ± 121.5	0.0422
Linoleic acid	C18:2n6c	34	4766 ± 28.6	3909.83 ± 159.5	0.0032
g-Linolenic acid	C18:3n6	34	5.33 ± 0.2	4 ± 0.0	0.0015
Linolenic acid	C18:3n3	34	106.33 ± 1.4	80.33 ± 2.3	<0.0001
Eicosenoic acid	C20:1n9	34	78.83 ± 3.6	68.83 ± 1.4	0.0297
Eicosadienoic acid	C20:2n6	34	70.5 ± 1.9	56.33 ± 1.5	0.0002
Eicosatrienoic acid	C20:3n6	34	62.17 ± 3.01	52.17 ± 2.1	0.218
Arachidonic acid	C20:4n6	34	738.17 ± 31.7	594 ± 16.1	0.0023
Docosahexaenoic acid	C22:6n3	34	242.33 ± 6.9	173.5 ± 4.1	<0.0001
Lignoceric acid	C24:0	34	6.83 ± 0.47	5.5.0 ± 0.2	0.0299
Lauric acid	C12:0	64	13.17 ± 0.3	6.33 ± 0.2	<0.0001
Myristic acid	C14:0	64	166.5 ± 3.5	135.67 ± 2.3	<0.0001
Palmitic acid	C16:0	64	6932.5 ± 68.6	7141.17 ± 30.1	0.0194
Palmitoleic acid	C16:1	64	619.7 ± 20.1	785.8 ± 9.3	<0.0001
Heptadecanoic acid	C17:0	64	58.33 ± 1.02	51.5 ± 0.3	<0.0001
Stearic acid	C18:00	64	2345 ± 13.8	2438 ± 36.5	0.039
Oleic acid	C18: 1n9c	64	11,579 ± 100.9	12,136 ± 111.0	0.004
Linoleic acid	C18:2n6c	64	5990.83 ± 89.3	5373.33 ± 43.7	0.0001
Eicosenoic acid	C20:1n9	64	78.33 ± 1.2	73.5 ± 1.4	0.0283
Eicosatrienoic acid	C20:3n6	64	61 ± 1.7	56 ± 0.3	0.0037
Lauric acid	C12:0	82	7.17 ± 0.0	8.67 ± 0.2	0.0024
Heptadecanoic acid	17:00	82	48.50 ± 1.3	55.5 ± 1.8	0.012
Nervonic acid	C24:1	82	10.17 ± 0.5	12.83 ± 0.6	0.0081

**Table 4:** Comparison of egg yolk crude protein values (%) from hens housed in CF and CC production systems (pools five yolks/system/week).

Hen Age	Production System		*p-*Value
(Week)	CF	CC	
34	13.80 ± 0.3	13.22 ± 0.0	0.368
64	14.69 ± 0.3	15.84 ± 0.3	0.1017
82	14.55 ± 0.2	13.87 ± 0.06	0.0346

## Data Availability

Data are contained within the article.

## References

[1] Molnár S., Szőllősi L. (2020). Sustainability and quality aspects of different table egg production systems: A literature review. Sustainability.

[2] Mottet A., Tempio G. (2017). Global poultry production: Current state and future outlook and challenges. World’s Poult. Sci. J..

[3] Castro F.L.S., Chai L., Arango J., Owens C.M., Smith P.A., Reichelt S., DuBois C., Menconi A. (2023). Poultry industry paradigms: Connecting the dots. J. Appl. Poult. Res..

[4] Gonzalez-Mora A.F., Rousseau A.N., Larios A.D., Godbout S., Fournel S. (2022). Assessing environmental control strategies in cage-free aviary housing systems: Egg production analysis and random forest modeling. Comput. Electron. Agric..

[5] Sosnówka-Czajka E., Herbut E., Skomorucha I. (2010). Effect of different housing systems on productivity and welfare of laying hens. Ann. Anim. Sci..

[6] Jones D.R., Cox N.A., Guard J., Fedorka-Cray P.J., Buhr R.J., Gast R.K., Abdo Z., Rigsby L.L., Plumblee J.R., Karcher D.M. (2015). Microbiological impact of three commercial laying hen housing systems. Poult. Sci..

[7] English M.M. (2021). The chemical composition of free-range and conventionally-farmed eggs available to Canadians in rural Nova Scotia. PeerJ.

[8] Janczak A.M., Riber A.B. (2015). Review of rearing-related factors affecting the welfare of laying hens. Poult. Sci..

[9] Løtvedt P., Fallahshahroudi A., Bektic L., Altimiras J., Jensen P. (2017). Chicken domestication changes the expression of stress-related genes in brain, pituitary and adrenals. Neurobiol. Stress.

[10] Henchion M., Moloney A.P., Hyland J., Zimmermann J., McCarthy S. (2021). Trends for meat, milk, and egg consumption for the next decades and the role played by livestock systems in the global production of proteins. Animal.

[11] Zhu L., Liao R., Wu N., Zhu G., Tu Y., Yang C. (2019). Integrating miRNA and mRNA expression profiles in plasma of laying hens associated with heat stress. Mol. Biol. Rep..

[12] Rozenboim I., Tako E., Gal-Garber O., Proudman J.A., Uni Z. (2007). The effect of heat stress on ovarian function of laying hens. Poult. Sci..

[13] Kim D.H., Lee Y.K., Lee S.D., Kim S.H., Lee S.R., Lee H.G., Lee K.W. (2020). Changes in production parameters, egg qualities, fecal volatile fatty acids, nutrient digestibility, and plasma parameters in laying hens exposed to ambient temperature. Front. Vet. Sci..

[14] Barrett N.W., Rowland K., Schmidt C.J., Lamont S.J., Rothschild M.F., Ashwell C.M., Persia M.E. (2019). Effects of acute and chronic heat stress on the performance, egg quality, body temperature, and blood gas parameters of laying hens. Poult. Sci..

[15] da Silva Pires P.G., Bavaresco C., Prato B.S., Wirth M.L., de Oliveira Moraes P. (2021). The relationship between egg quality and hen housing systems systematic review. Livest. Sci..

[16] Samiullah S., Omar A.S., Roberts J., Chousalkar K. (2017). Effect of production system and flock age on eggshell and egg internal quality measurements. Poult. Sci..

[17] Dikmen B.Y., Ipek A.Y.D.I.N., Şahan Ü., Petek M.E.T.I.N., Sözcü A. (2016). Egg production and welfare of laying hens kept in different housing systems (conventional, enriched cage, and free range). Poult. Sci..

[18] Philippe F.X., Mahmoudi Y., Cinq-Mars D., Lefrançois M., Moula N., Palacios J., Pelletier F., Godbout S. (2020). Comparison of egg production, quality and composition in three production systems for laying hens. Livest. Sci..

[19] Haugh R.R. (1937). The Haugh unit for measuring egg quality. United States Egg Poult. Mag..

[20] (2014). Animal and Vegetable Fats and Oils—Gas Chromatography of Fatty Acid Methyl esters—Part 1: Guidelines on Modern Gas Chromatography of Fatty Acid Methyl esters.

[21] (2017). Animal and Vegetable Fats and Oils—Gas Chromatography of Fatty Acid Methyl Esters—Part 2: Preparation of Methyl Esters of Fatty Acids.

[22] Mooijman K.A. (2018). The new ISO 6579-1: A real horizontal standard for detection of Salmonella, at last!. Food Microbiol..

[23] Mikoleit M.L. (2010). WHO Global Foodborne Infections Network. A WHO Network Building Capacity to Detect, Control, and Prevent Foodborne and Other Enteric Infections from Farm to Table” Laboratory Protocol: “Isolation of Salmonella and Shigella from Faecal Specimens”.

[24] Yenice G., Kaynar O., Ileriturk M., Hira F., Hayirli A. (2016). Quality of eggs in different production systems. Czech J. Food Sci..

[25] Alig B.N., Malheiros R.D., Anderson K.E. (2023). Evaluation of Physical Egg Quality Parameters of Commercial Brown Laying Hens Housed in Five Production Systems. Animals.

[26] Schuck-Paim C., Negro-Calduch E., Alonso W.J. (2021). Laying hen mortality in different indoor housing systems: A meta-analysis of data from commercial farms in 16 countries. Sci. Rep..

[27] Sinclair M., Lee N.Y., Hötzel M.J., de Luna M.C.T., Sharma A., Idris M., Islam M.A., Iyasere O.S., Navarro G., Ahmed A.A. (2022). Consumer attitudes towards egg production systems and hen welfare across the world. Front. Anim. Sci..

[28] de Luna M.C.T., Yang Q., Agus A., Ito S., Idrus Z., Iman R.H., Jattuchai J., Lane E., Nuggehalli J., Hartcher K. (2022). Cage egg producers’ perspectives on the adoption of cage-free systems in China, Japan, Indonesia, Malaysia, Philippines, and Thailand. Front. Vet. Sci..

[29] Racevičiūtė-Stupelienė A., Vilienė V., Bliznikas S., Šašytė V., Nutautaitė M. (2023). The relationship between different laying hen housing systems in Lithuania and egg production quality and chemical composition. Cogent Food Agric..

[30] Roberts J.R., Chousalkar K.K. (2014). Effect of production system and flock age on egg quality and total bacterial load in commercial laying hens. J. Appl. Poult. Res..

[31] Soler Castillo R., Mínguez Balaguer C., Ibanez Sanchis C., Bueso Rodenas J. (2022). Egg size and quality of hens housed in three different group sizes. J. Appl. Anim. Res..

[32] Şekeroğlu A., Duman M., Tahtalı Y., Yıldırım A., Eleroğlu H. (2014). Effect of cage tier and age on performance, egg quality and stress parameters of laying hens. S. Afr. J. Anim. Sci..

[33] Kucukkoyuncu E., Okur A.A., Tahtabiçen E., Korkmaz F., Samli H.E. (2017). Comparing the quality of free range and battery cage eggs. Eur. Poult. Sci./Arch. Für Für Geflügelkunde.

[34] Sokołowicz Z., Krawczyk J., Dykiel M. (2018). Effect of alternative housing system and hen genotype on egg quality characteristics. Emir. J. Food Agric..

[35] Speake B.K., Murray A.M.B., Noble R.C. (1998). Transport and transformations of yolk lipids during the development of the avian embryo. Prog. Lipid Res..

[36] Marelli S.P., Madeddu M., Mangiagalli M.G., Cerolini S., Zaniboni L. (2021). Egg Production Systems, Open Space Allowance and Their Effects on Physical Parameters and Fatty Acid Profile in Commercial Eggs. Animals.

[37] Lordelo M., Fernandes E., Bessa R.J.B., Alves S.P. (2017). Quality of eggs from different laying hen production systems, from indigenous breeds and specialty eggs. Poult. Sci..

[38] Whelan J., Fritsche K. (2013). Linoleic acid. Adv. Nutr..

[39] Orczewska-Dudek S., Pietras M., Puchała M., Nowak J. (2020). Camelina sativa oil and camelina cake as sources of polyunsaturated fatty acids in the diets of laying hens: Effect on hen performance, fatty acid profile of yolk lipids, and egg sensory quality. Ann. Anim. Sci..

[40] Tomaszewska E., Muszyński S., Arczewska-Włosek A., Domaradzki P., Pyz-Łukasik R., Donaldson J., Świątkiewicz S. (2021). Cholesterol Content, Fatty Acid Profile and Health Lipid Indices in the Egg Yolk of Eggs from Hens at the End of the Laying Cycle, Following Alpha-Ketoglutarate Supplementation. Foods.

[41] Hidalgo A., Rossi M., Clerici F., Ratti S. (2008). A market study on the quality characteristics of eggs from different housing systems. Food Chem..

[42] Mika A., Van Treuren W., González A., Herrera J.J., Knight R., Fleshner M. (2015). Exercise is more effective at altering gut microbial composition and producing stable changes in lean mass in juvenile versus adult male F344 rats. PLoS ONE.

[43] Kawamura N., Yokoyama R., Takaya M., Ono R., Goto T. (2023). The combined effect of feed and housing system affects the free amino acid content of egg yolk and albumen in brown layer chickens. J. Poult. Sci..

[44] Koutsoumanis K., Allende A., Alvarez-Ordóñez A., Bolton D., Bover-Cid S., Chemaly M., Herman L., Hilbert F., Lindqvist R., EFSA Panel on Biological Hazards (EFSA BIOHAZ Panel) (2019). *Salmonella* control in poultry flocks and its public health impact. EFSA J..

[45] Sharma M.K., McDaniel C.D., Kiess A.S., Loar II R.E., Adhikari P. (2022). Effect of housing environment and hen strain on egg production and egg quality as well as cloacal and eggshell microbiology in laying hens. Poult. Sci..

[46] Mollenhorst H., Van Woudenbergh C.J., Bokkers E.G.M., De Boer I.J.M. (2005). Risk factors for Salmonella enteritidis infections in laying hens. Poult. Sci..

